# Micro-abnormalities in the retina and choroid induced by anti-CTLA4 treatment

**DOI:** 10.1038/s41598-023-33582-5

**Published:** 2023-04-18

**Authors:** Ryo Mukai, Yuki Tajika, Kazuma Saito, Hideo Akiyama

**Affiliations:** 1 Department of Ophthalmology, Gunma University Graduate School of Medicine, 3-35-15 Showa-cho, Maebashi, Gunma 371-8511 Japan; 2 Department of Anatomy, Gunma University Graduate School of Medicine, Maebashi, Japan

**Keywords:** Neuroscience, Medical research, Pathogenesis

## Abstract

Anti-Cytotoxic T-Lymphocyte Associated protein 4 agents, such as ipilimumab, are widely applied to various cancers. However, they cause immune-related adverse effects throughout the body, including the eye. This study examined whether ipilimumab induces retinal and choroidal abnormalities in rodents, and investigated potential underlying mechanisms. Female wild-type mice were injected with ipilimumab three times/week for 5 weeks. The mice underwent optical coherence tomography (OCT) on the first day of the 6th week. Retinal function and morphology were evaluated by light microscopy, immunohistochemistry and electroretinography (ERG). On OCT, the lines indicating the ellipsoid and interdigitation were obscure in treated mice, suggesting outer retina destruction. Haematoxylin–eosin staining revealed destruction, shortening, and outer segment vacuolization. Treated mice exhibited weaker, fragmented rhodamine peanut agglutinin staining in outer photoreceptor structures. The choroid of treated mice showed severe infiltration of CD45-positive cells. In addition, CD8-positive cells invaded into the outer retina. On ERG, rod, maximum responses of combined rods and cones, and cone response wave amplitudes were significantly reduced in treated mice. Ipilimumab may induce impairments in outer photoreceptor architecture accompanied with CD8- positive infiltration in the retina and CD45-positive cell infiltration in the choroid, which may contribute to retinal function deterioration.

## Introduction

Anti-Cytotoxic T-Lymphocyte Associated protein 4 (CTLA4) agents, such as ipilimumab represent a major class of drugs used in the treatment of incurable malignant melanoma, along with inhibitors of programmed cell death protein 1 (PD1) and programmed death ligand 1^1^. Recently, anti-CTLA4 agents have been widely applied to a broad range of cancers, and the use of this drug seems to have increased worldwide^2^. However, given their function as immune checkpoint inhibitors, anti-CTLA4 agents can cause immune-related adverse effects throughout the body^3^ and within the eye^4^.

During a phase 1 study of ipilimumab for the treatment of malignant melanoma, adverse effects, such as uveitis (including cases of iritis, vitritis, and diseases involving posterior serous retinal detachment, such as Vogt–Koyanagi–Harada [VKH] disease) were observed within 2 weeks after first administration^5^. Since this trial, cases of uveitis occurring during administration of anti-CTLA4 agents have frequently been reported worldwide^6–8^. One patient with malignant melanoma who was treated with an anti-CTLA4 agent at our institution exhibited bilateral serous retinal detachments (SRDs) 22 days after treatment. In this case, SRD was accompanied by elongation of the outer photoreceptor segment, both within and beyond the area of SRD, and SRD persisted without complete resolution^9^. A similar case was reported in a patient treated with PD1 inhibitors, who experienced poor recovery of visual function^10^. These cases highlight the need to elucidate mechanisms underlying such adverse effects to ensure maintenance of visual function in patients treated with anti-CTLA4 agents.

Given that anti-CTLA4 agents suppress the function of the retinal pigment epithelium (RPE), we hypothesized that they would also induce inflammation in the retina and choroid. In the present study, we investigated this hypothesis by applying anti-CTLA4 treatment in a rodent model.

## Materials and methods

### Animals

Nine-week-old female C57BL/6J mice (CLEA Japan Inc., Tokyo, Japan) were used for this study. All animal procedures in this study adhered to the ARRIVE guidelines and the Association for Research in Vision and Ophthalmology Statement for the Use of Animals in Ophthalmic and Vision Research. They were approved by the Animal Care and Use Committee of Gunma University Graduate School of Medicine.

### Induction of inflammation via injection of a CTLA4 blocking antibody

**Figure 1:**
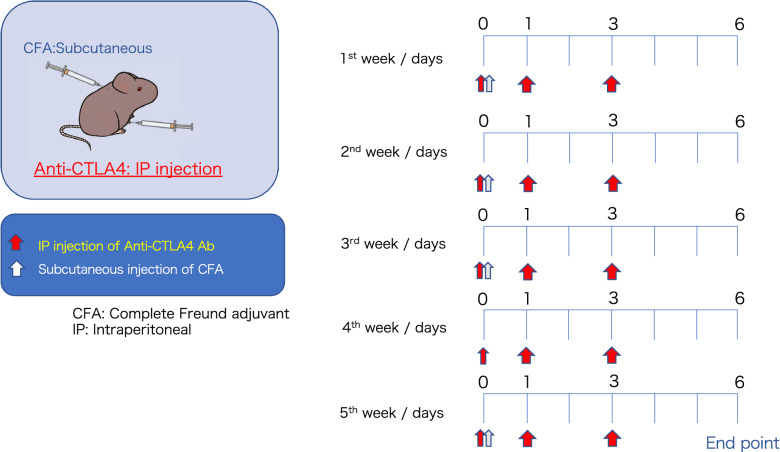
Schedule of anti-CTLA4 and complete Freund adjuvant injections in mice. IP: intraperitoneal.

Inflammation was induced in the retina and choroid, as previously described^11^. Briefly, animals were intraperitoneally injected with 300 μg/100 μL of anti-CTLA4 antibody (Bio X Cell, Lebanon, NH, USA; InvivoMab 9D9; Cat. #BE0164), which was adjusted with phosphate-buffered saline (PBS), three times weekly (days 0, 1, and 3) for 5 weeks. To enhance the immune response, 100 μL of complete Freund’s adjuvant (Rocklands Immunochemicals Inc., Pottstown, PA, USA; D614-0050) was injected on the first day of the weekly protocol, except during the fourth week (Fig. 1).

### Optical coherence tomography

Mice were anesthetized with an intraperitoneal injection of ketamine (80 mg/kg) and xylazine (10 mg/kg) and the pupils were dilated with topical drops of phenylephrine (5%) and tropicamide (0.5%). Optical coherence tomography (OCT) B-scan images, each composed of 25 averaged scans obtained using eye tracking, were obtained around the optic nerve head (Spectralis HRA + OCT; Heidelberg Engineering, Heidelberg, Germany). Mice were placed on the device such that the fundus was in focus. OCT images were acquired using a non-contact type lens, which attached to the HRA-OCT.

### Histological analyses of retinal and choroidal tissue

For qualitative analysis of the retina and choroid, we analysed whole-eye cryosections obtained 5 weeks after injection. The eyes were embedded in optimal cutting temperature compound (4583; Tissue Tek; Sakura Finetek, Torrance, CA, USA) immediately after enucleation under general anaesthesia using ketamine and xylazine and perfused with a fixative solution (4% paraformaldehyde [PFA] in PBS) and were frozen via submersion in isopropanol pooled in a small metal jar chilled in liquid nitrogen. Retinal and choroidal cryosections (10-μm-thick) were stained with haematoxylin–eosin (HE) for analysis. For analysis of choroidal flatmount, eyes were enucleated after perfusion fixation using 4% PFA and choroidal flatmount was prepared. The images of choroidal flatmount were taken using a stereoscopic microscope (SZX7; Olympus, Tokyo, JAPAN) with a camera (EOSR, Canon, JAPAN) accompanied with a high magnification lens (MP-E65F2, Canon, JAPAN).

### Immunohistochemistry

Eyes were placed in a fixative consisting of 4% PFA in PBS for 1 h at room temperature immediately after enucleation, following which 10-μm-thick cryosections of the retina and choroid were prepared as described above. The slices were placed in blocking buffer (PBS containing 2% bovine serum albumin [BSA]) for 10 min at room temperature and subsequently incubated with rhodamine-labelled peanut agglutinin (PNA; Vector Laboratories, Burlingame, CA, USA; RL-1072, 1:500) and DAPI (Sigma, St Louis, MO, USA; D9542, 1:200) in blocking buffer for 60 min at room temperature. The retina sections were then washed three times for 5 min in PBS. For CD45 and CD 8 staining, retinas were incubated with anti-mouse CD45 polyclonal antibody (R&D Systems, Minneapolis, MN, USA; AF-114SP 1:300) and CD8 monoclonal antibody (Abcom, Cambridge, MA, USA, ab22378 1:1000) in blocking buffer overnight at 4 °C, washed three times for 5 min in PBS containing 1% BSA, and incubated with Alexa Flour 488 (Alexa Fluor 488-conjugated donkey anti-goat IgG; Abcam, Cambridge, MA, USA; 150,129, 1:500) or Alexa Flour 594 (Alexa Fluor 594-conjugated donkey anti-rat IgG; Abcam, Cambridge, MA, USA; 21,209, 1:500) for 1 h at room temperature. In contrast, a choroidal flat mount was prepared after perfusion fixation. The flat mount was placed in blocking buffer (PBS containing 5% normal donkey serum, 0.1% Tween20, and 0.01% Triton) for 1 h at room temperature. The samples were stained for zonula occludens 1 (ZO1; Thermo Fisher Scientific, Waltham, MA, USA, 61–7300, 1:200) and incubated overnight at 4 °C, following which they were washed three times with PBS for 5 min and incubated with Alexa Flour 488 (Alexa Fluor 488-conjugated donkey anti-rabbit IgG, Jackson ImmunoReseach, West Grove, PA, USA; 1:500) for 1 h at room temperature. A fluorescence microscope (LSM880; Zeiss Microscope, Jena, Germany), equipped with a computer-driven motorized stage controlled by image analysis software (Zen2 2015, Zeiss Microscope), was used to capture images (with 40 × , 63 × objectives, with oil). To reconstruct recorded images, we used FIJI using Z-projection (Max).

### Terminal deoxynucleotidyl transferase dUTP nick end (TUNEL) labelling and quantification of retinal cell death

Five weeks after the first anti-CTLA4 injection, the eyes were enucleated and frozen in an optimal cutting temperature compound, using isopropanol chilled in liquid nitrogen. The eyes were sectioned into 14-µm-thick sections for TUNEL labelling, which was performed using a Sigma TUNEL kit (Sigma, S7111) in accordance with the manufacturer’s instructions. The sections were cover-slipped with DAPI-containing medium. Using a 40 × objective, a DAPI image was taken at the midpoint of the retina. The DAPI image was then overlaid on an image of the green fluorescent protein channel (TUNEL-positive cells) to confirm that both were collocated.

### Electroretinogram

Five weeks after the first anti-CTLA4 injection, electroretinograms (ERG) were recorded in mice that had or had not received anti-CTLA4 injections, using a Ganzfeld dome, an acquisition system (PuREC, Bunkyo, Japan), and an LED stimulator (LS-100; Mayo Corp., Inazawa, Japan). Following dark adaptation of the mice overnight, the pupils were dilated using 0.5% phenylephrine and 0.5% tropicamide eye drops under general anaesthesia. In this study, full-field ERG included eleven ERG protocols: A series of white flashes (− 4.0, − 3.0, − 2.0, − 1.0, 0, and + 1.0 log cd˙s/m^2^) were applied under dark adaptation, followed by a series under light adaptation (0, 0.5, 1, and 1.5 log cd˙s/m^2^). A positive contact lens electrode was also used for analysis at a 12-Hz flicker.

### Statistical analysis

Two-way analyses of variance were used to compare a-wave, b-wave, and cone amplitudes between the anti-CTLA4 and control groups. Other factors were compared using unpaired, two-tailed *t*-tests. Statistical significance was set at *P* < 0.05. All analyses were performed using GraphPad Prism version 9.3.1 (GraphPad Software, San Diego, CA, USA).

## Results

### Micro-abnormalities of the outer retina on OCT and impairments in outer retinal structure and choroidal thickening

**Figure 2:**
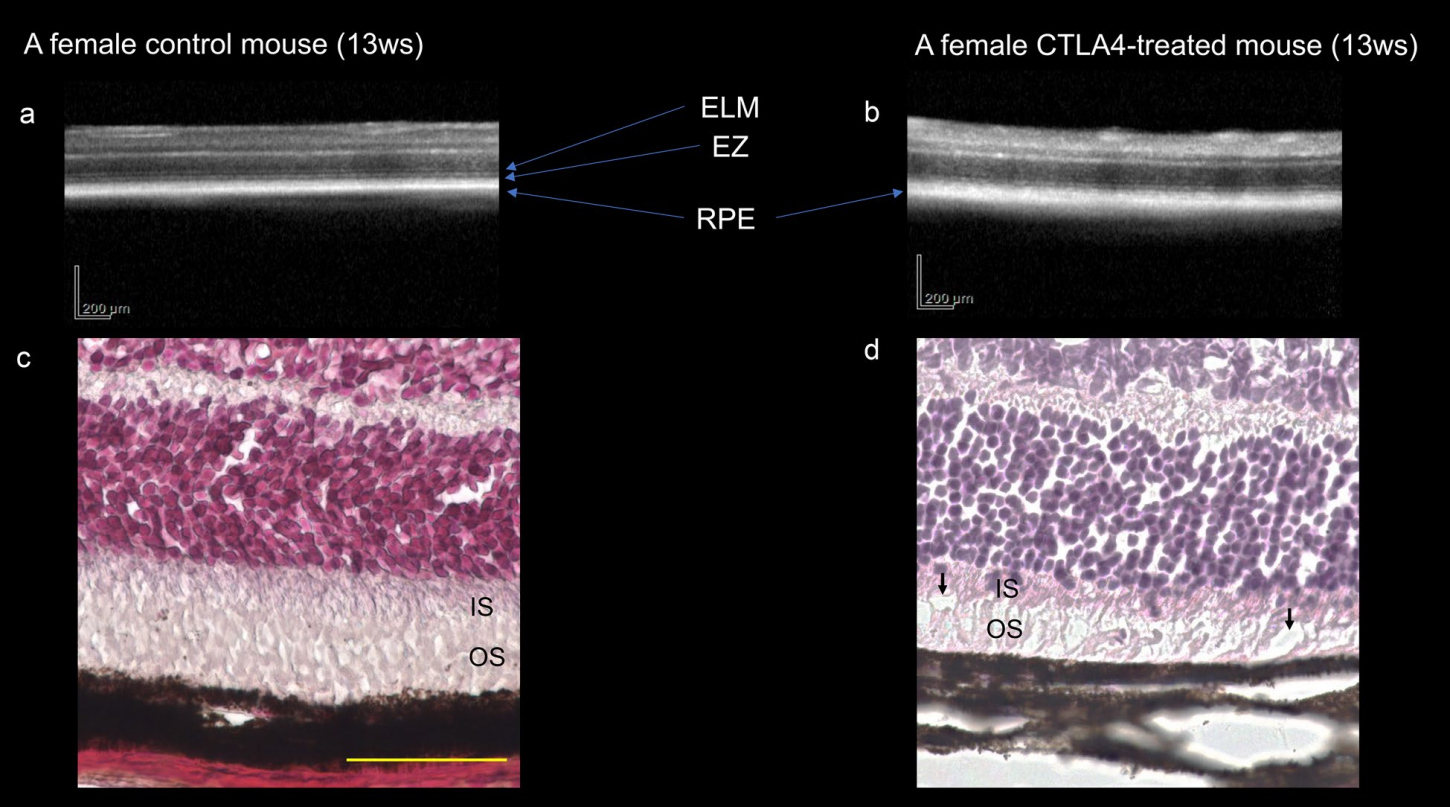
(**A**), (**B**) Retinal structure in control and anti-CTLA4-treated eyes examined using optical coherence tomography. Treated eyes exhibited an obscured external limiting membrane (ELM) and ellipsoid zone (EZ) as compared to control eyes. (**C**), (**D**) Haematoxylin and eosin staining for control and anti-CTLA4-treated eyes. Treated eyes exhibited destruction of outer photoreceptor segments and choroidal thickening. Black arrows indicated vacuolisations. Scale bar: 50 μm.

Five weeks after the first anti-CTLA4 injection, OCT revealed obscurity of both the outer limiting membrane line and ellipsoid zone around the optic nerve head in experimental mice (n = 4), although the outer retinal microstructures were preserved in control mice (n = 4) (Fig. 2A,B). The thickness of inner and outer nuclear layer (ONL) was 25.8 ± 1.3 μm and 59.1 ± 0.6 μm in the control group, respectively and 25.9 ± 0.7 μm and 57.8 ± 1.0 μm in the anti-CTLA4 treated eyes, respectively. There was no significant difference in the thicknesses between the groups (*P* = 0.9182 and 0.0652, respectively).After OCT examination, the mice were sacrificed, and changes in retinal morphology were analysed using HE staining. Impairment and fragmentation of both the inner and outer segments were observed in retinal samples obtained from anti-CTLA4-treated mice. In the outer portion of the retina, vacuolization was accompanied by impairment of inner and outer segment. In addition, thickening of the choroid was prominent in the retinas of mice treated with anti-CTLA4 (n = 3 in the control and treated groups, respectively) (Fig. 2C,D). The HE sections revealed no prominent depigmentation in the choroid or RPE.

### Limited depigmentation of RPE

**Figure 3:**
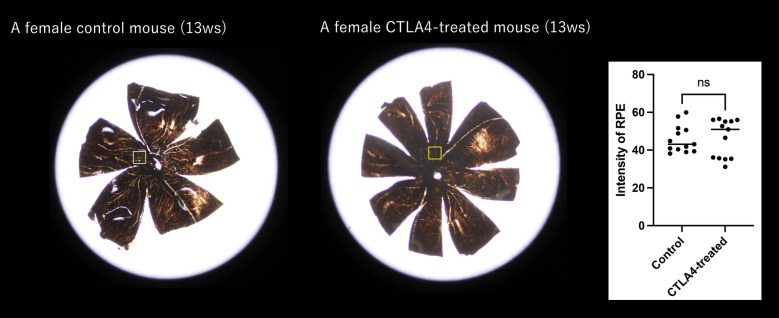
The mean intensity of choroidal flatmount at each lobule (100 × 100 pixels) in the control group and CTLA4 treated eyes was 45.88 ± 7.26 and 46.35 ± 9.99, respectively. There was no significant difference in the intensity of RPE. This result suggested that the depigmentation of the RPE was highly limited.

The mean intensity of choroidal flatmount at each lobule (100 × 100 pixels) in the control group and CTLA-4 treated eyes was 45.88 ± 7.26 and 46.35 ± 9.99, respectively. There was no significant difference in the intensity of RPE. This result suggested that the depigmentation of the RPE was highly limited (Fig. 3).

### Immunohistochemistry

#### Impairment of inner and outer segment of photoreceptors

**Figure 4:**
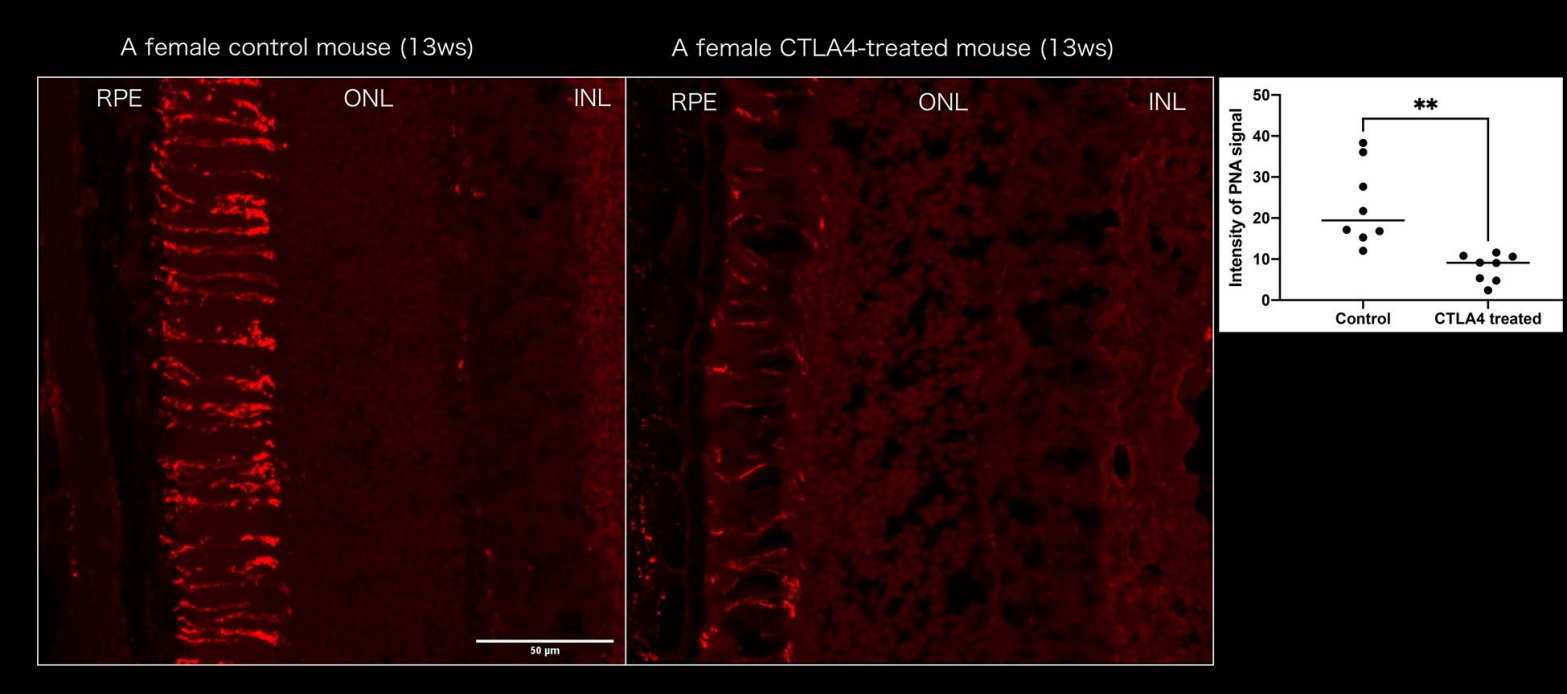
Peanut agglutinin (PNA) staining in control and anti-CTLA4-treated eyes (n = 4 in each group). Control eyes exhibited linear staining along the outer segment. In contrast, partial or fragmented staining was observed in treated eyes. The intensity of PNA staining was significantly lower in treated eyes than in control eyes. Scale bar: 50 μm. **P > 0.01. RPE: retinal pigment epithelium, ONL: Outer nuclear layer, INL: Inner nuclear layer.

First, rhodamine-labelled PNA was applied to the retina, which revealed clear breakdown of the inner and outer segments throughout the outer retina. The PNA intensities of the outer retina in the control and treated with anti-CTLA-4 mice were 7.96 ± 3.36 and 23.12 ± 9.86, respectively, with significantly weak staining in the treated eyes (Fig. 4).

#### Limited number of apoptotic cells in ONL

**Figure 5:**
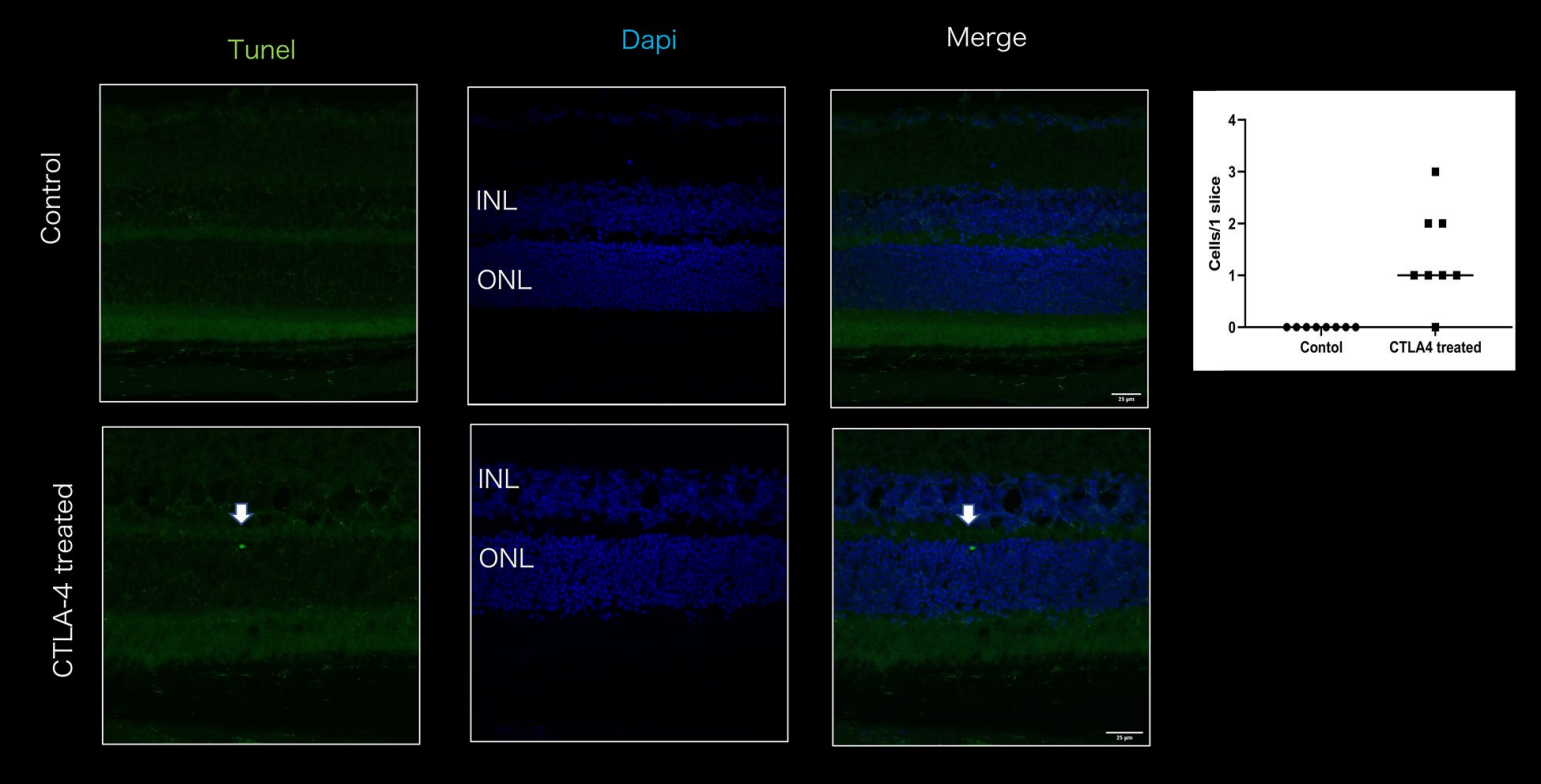
TUNEL staining in control and anti-CTLA4-treated eyes. Very limited numbers of TUNEL-positive cells were detected in treated eyes (n = 4 in each group). ONL: Outer nuclear layer, INL: Inner nuclear layer. The white arrows indicated the TUNEL positive cell in treated eye.

TUNEL staining was then performed to determine whether such impairments were accompanied by photoreceptor cell death. In the experimental group, only one to three TUNEL-positive cells (mean: 1.3 ± 0.9 cells) were detected in retinal slices of the whole eye, although no TUNEL-positive cells were detected in the control group (Fig. 5).

#### Limited impairment of RPE

**Figure 6:**
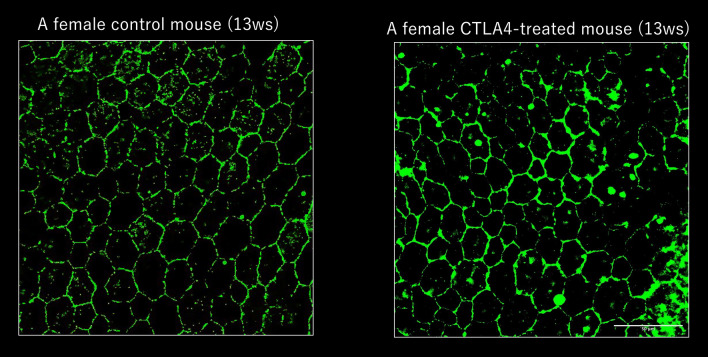
ZO1 staining in control and anti-CTLA4-treated eyes. Misalignment and discontinuous staining were observed in treated eyes (n = 3 in each group).

Morphological changes in the RPE were also analysed based on staining for ZO1. Heterogenous ZO1 staining and misaligned marginal staining of the RPE were more prominent in anti-CTLA4 mice than in control mice (n = 3 in each group) (Fig. 6).

#### Invasion of CD45 positive cells in the retina and choroid and CD8 positive cells in the retina

**Figure 7:**
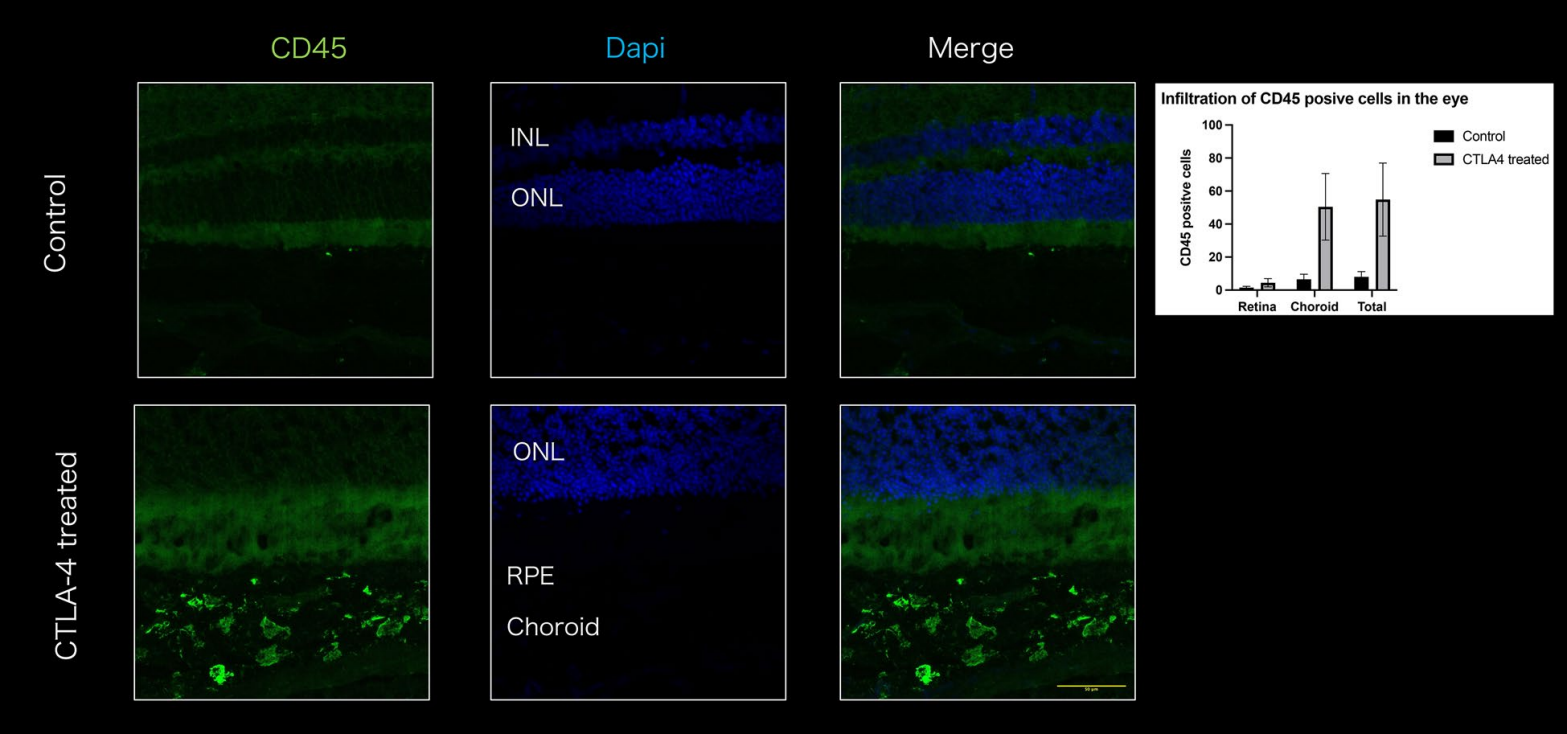
CD45 staining in control and anti-CTLA4-treated eyes (n = 7 in each group). Choroidal infiltration of round or ameboid-like CD45-positive cells was observed in treated eyes. There was a significant difference in the number of CD45-positive cells in the choroid between control and treated eyes. On the other hand, very limited CD45-positive staining was observed in the retina. RPE: retinal pigment epithelium, ONL: Outer nuclear layer, INL: Inner nuclear layer.

**Figure 8:**
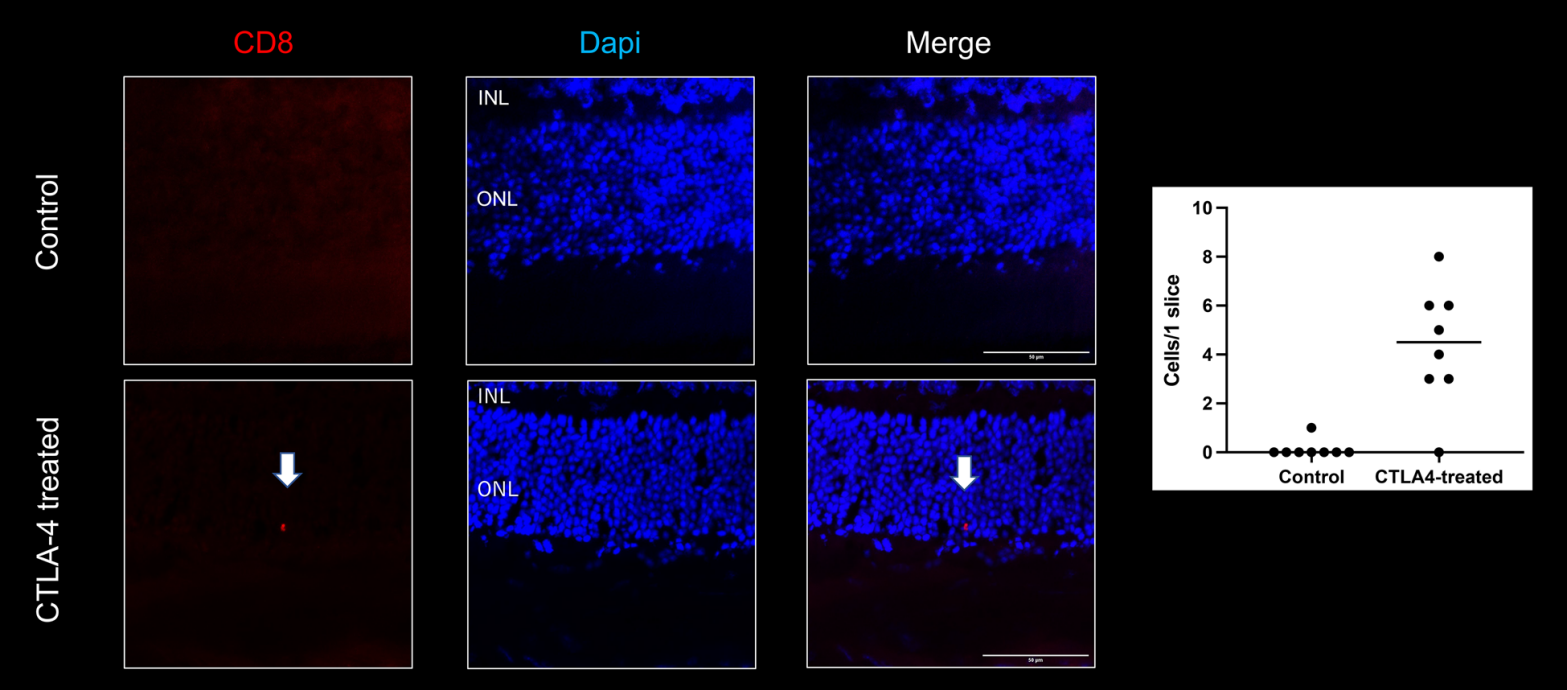
CD8 staining in control and anti-CTLA4-treated eyes (n = 4 in each group). CD8-positive cells were observed in treated eyes, by contrast very limited those cells were detected in the control. There was a significant difference in the number of CD8-positive cells in the retina between control and treated eyes. ONL: Outer nuclear layer, INL: Inner nuclear layer.

Finally, to clarify the immune response to anti-CTLA4 treatment in the retina, CD45 and CD8 staining were performed to examine changes in the retina and choroid in mice receiving anti-CTLA4 treatment. Marked invasion of CD45-positive cells was observed in the choroidal tissue of mice treated with anti-CTLA4 (mean number was 50.4 ± 20.2 cells), and the number of cells detected was significantly higher than that in the control group (mean number was 6.5 ± 3.2 cells). In contrast, very few CD45-positive cells were detected in both normal and treated retinas (Fig. 7). We also assessed invasion of CD 8 positive cells. In the retina treated with anti-CTLA4, 4.3 ± 2.4 CD 8 positive cells were detected; by contrast, only 0.1 ± 0.4 cells were detected in the control retina (Fig. 8).

### Electroretinogram findings

**Figure 9:**
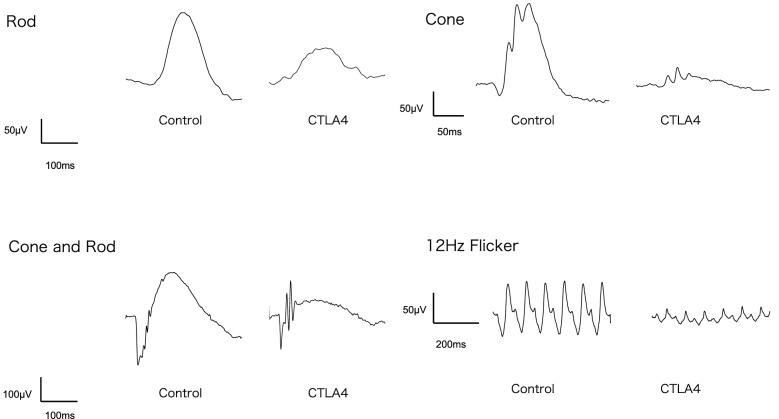
Representative a-wave, b-wave, cone, and 12-Hz flicker responses for control and treated eyes. For all waves, the amplitudes were lower for treated eyes than for control eyes. Despite the lower amplitudes, wave forms were preserved (n = 5 in each group).

**Figure 10:**
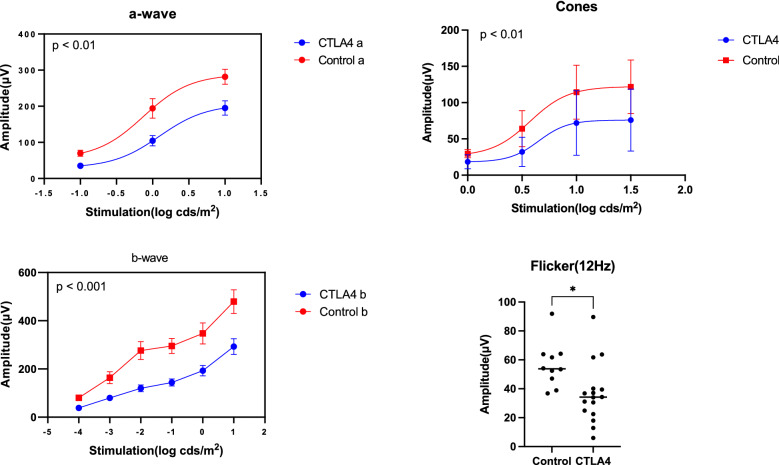
Mean responses for the a-wave, b-wave, cones, and 12-Hz flicker tests for control and treated eyes (n = 5 in each group). In the analysis of whole waves, the mean amplitudes were significantly lower for treated eyes than for control eyes (*P* < 0.01, *P* < 0.001, and *P* < 0.01, respectively).

Whole amplitudes for a-wave, b-wave, and cones were significantly lower in the anti-CTLA4 group than in the control group after 5 weeks of treatment (*P* < 0.01, *P* < 0.001, and *P* < 0.01, respectively, shown in Table 1). In addition, the maximum amplitude of the 12-Hz flicker was significantly lower in the anti-CTLA4 group (38.453 ± 23.274 V) than in the control group(80.471 ± 32.816) (*P* < 0.05). The amplitudes of the rods, maximum, cones, and flicker responses are summarized in Table 1 and Figs. 9 and 10. Representative electroretinogram waves are shown in Fig. 9.

**Table 1:** Mean responses (μV) for the a-wave, b-wave, cones, and 12-Hz flicker tests for control and treated eyes (n = 5 in each group).

Averaged amplitude		Stimulation (log cds/m^2^)	Controls	CTLA4 treated	*P* value
Dark adaptation	a-wave	− 1	69.684 ± 27.077	35.197 ± 20.377	0.0011
		0	194.333 ± 85.923	104.414 ± 55.772	0.0034
		1	281.616 ± 65.368	195.166 ± 78.976	0.008
	b-wave	− 4	80.471 ± 32.803	38.453 ± 23.261	0.0008
		− 3	163.957 ± 78.632	79.989 ± 38.500	0.0012
		− 2	276.167 ± 1 17.513	119.953 ± 57.774	0.0001
		− 1	295.241 ± 97.916	143.653 ± 57.726	< 0.0001
		0	347.262 ± 137.451	192.749 ± 85.987	0.0017
		1	479.250 ± 154.587	292.653 ± 130.706	0.003
Light adaptation		0	29.590 ± 5.616	18.422 ± 9.698	0.0055
		0.5	64.158 ± 24.909	32.024 ± 20.167	0.0014
		1	114.380 ± 37.196	71.987 ± 44.556	0.0193
		1.5	121.718 ± 37.056	75.883 ± 42.771	0.0101
	12 Hz Flicker	0.5	80.471 ± 32.816	38.453 ± 23.274	0.0152

## Discussion

In this study, we investigated whether anti-CTLA4 agent, induces retinal and choroidal impairments in rodents. Our analysis revealed that repeated anti-CTLA4 injections induced micro-abnormalities in the retina and retinal pigment epithelium. Chief impairments were observed in structures of the outer retina, including the photoreceptors. Infiltration of CD45-positive cells in the choroid and CD8-positive cells in the retina were also observed. Deterioration of retinal function was observed based on electroretinogram responses for rods, cones, and maximum response of combined rods and cones. Overall, these results suggested that anti-CTLA4 treatment can induce impairments of outer retina due to infiltration of CD8-positive cells. In addition, CD45-positve cells invaded in the choroid. These results suggested that anti-CTLA4 might induced disturbance in the retina via CD8 positive cells and invasion of CD 45 positive cells in the choroid possibly contribute to retrograde breakdown of the inner and outer segments of the photoreceptors, leading to deficits in retinal function.

Iwama et al. were the first to report anti-CTLA4-induced hypophysitis in a mouse model^11^. They observed infiltration of immune cells, such as monocytes, CD45-positive cells, and macrophages, in the pituitary gland after the administration of anti-CTLA4^11^. The authors also reported deposition of complement components in the tissue. In our study, mice treated with anti-CTLA4 exhibited distortion of the outer retinal structure and misalignment of the RPE.

In this study, we had originally utilized the same duration and frequency of injections, although the dose of anti-CTLA4 injections was increased to 300 µg given that no morphological changes were observed following injections of 100 µg (corresponding to the equivalent amount used for humans in a previous study, n = 5) or 200 µg (n = 10) in our preliminary experiments in which we used OCT and HE investigations. Few TUNEL-positive cells were observed in the outer and inner nuclear layers or in the retinal ganglion cell layer in our study, suggesting that both RPE dysfunction and impairment of the outer and inner segment of photoreceptors rather than photoreceptor cell death was associated with deterioration of the outer retinal morphology.

Several studies have indicated that VKH disease is associated with histopathological changes in the choroid^12,13^. Infiltration of plasma cells and lymphocytes and the presence of CD3-positive cells in the choroid have been observed in HE and immunohistochemistry sections from human patients. Lymphocyte infiltration has also been observed in choroidal tissue from canine models with experimentally induced VKH disease^14,15^. In addition, partial depigmentation of choroidal melanocytes has been observed in deep choroidal tissue from human patients. In the current study, we observed strong infiltration of CD45-positive cells in the choroid, which seems to correspond to previous choroidal histological analyses of VKH disease. We attempted to identify the type of infiltrating CD45-positive cells by immunostaining for CD8, and IBA-1, but these attempts were unsuccessful. CD8-positive cells in the retina treated with anti-CTLA4 were observed in this study. In previous report of rat VKH experimental model, inflammatory cells were detected in the subretinal fluid. We believe that those infiltration of CD8-positive cells in the retina potentially did harm on the outer retina directly.

Our results of HE sections also suggested choroidal thickening at 5 weeks after anti-CTLA4 treatment. However, HE sections revealed no prominent depigmentation in the choroid or RPE, despite partial disturbances in the RPE based on ZO-1 staining. In addition, no mice in our study exhibited skin depigmentation. Thus, we believe that anti-CTLA4 administration induces activation and infiltration of inflammatory cells in the choroid, without prominent destruction of pigmentary cells.

Analysis of the ERG findings revealed strong reductions in rods, cones, and maximum responses of combined rods and cones, including flicker responses, in mice treated with anti-CTLA4. These results suggested that damage in this model was limited to the outer retinal microstructure and choroid, while the inner structures, at least thickness of INL, were preserved. A previous study reported worsening of scotopic and photopic ERG responses in cases with VKH disease, and such reductions appeared to be correlated with the severity of abnormalities in the fundus^16^. Another study indicated that these reductions seemed to recover in most cases of VKH disease over a 12-month period^17^. Interestingly, choroidal thickening at baseline and improvements in choroidal thickening during the clinical course may be correlated with decrease of ERG amplitudes. Even in cases with commotio retina, which showed damage of the inner and outer segment of the retina, the amplitude of maximum response of cones and rods was reported to decrease^18^. Our model exhibited reduced scotopic and photopic responses accompanied with invasion of inflammatory cells in the choroid, suggesting that the decreased amplitude could associate with outer retinal morphological change like commotio and choroidal pathogenesis in this model like VKH disease. Moreover, even though the thickness of INL was preserved, it was possible that the connection via synapses was destroyed during treatment. Further studies are required to clarify the status of synapses.

Clinically, subretinal fluid accumulation and macular oedema can occur following anti-CTLA4 treatment, with some patients also experiencing deterioration of visual function^5,9,10^. In this study, we utilized OCT to examine the emergence of macular oedema during anti-CTLA4 injection; however, no exudative changes were detected in the retinas of experimental mice. Nonetheless, functional deterioration of the retina was identified based on ERG findings. Thus, long-lasting macular oedema may cause destruction of the outer retinal morphology, which may lead to visual impairment in humans. In contrast, choroidal inflammation can alter the function of normal RPE, which could potentially lead to destruction of the outer retina, which may in turn cause disturbances of retinal function in rodents.

Anti-CTLA4 treatment has been reported to affect the eye as well as accessory tissues^4^, with the potential to induce optic neuropathy, myasthenia gravis, keratitis, and corneal injury during administration. In this study, no prominent injuries were observed in the cornea, conjunctiva, or optic nerves of mice following anti-CTLA4 treatment.

In conclusion, our results demonstrated that anti-CTLA4 Ab injection could induce both anatomical and functional impairments in the mouse retina, which accompanied with infiltration of CD45-positive cells in the choroid and CD-8 positive cells in the retina. The current findings could help understand the underlying pathogenesis in which incidental ocular adverse effects occurred in patients who underwent CTLA4 treatment.

## Data Availability

The dataset used in this article is available upon a formal and reasonable request from the corresponding author.
